# Significant Association between Subclinical Left Cardiac Dysfunction and Liver Stiffness in Metabolic Syndrome Patients with Diabetes Mellitus and Non-Alcoholic Fatty Liver Disease

**DOI:** 10.3390/medicina59020328

**Published:** 2023-02-09

**Authors:** Alexandru Apostu, Daniel Malita, Sergiu-Florin Arnautu, Mirela-Cleopatra Tomescu, Dan Gaiță, Alina Popescu, Ruxandra Mare, Ramona Gidea, Diana-Aurora Arnautu

**Affiliations:** 1 Multidisciplinary Heart Research Center of the Victor Babes University of Medicine and Pharmacy, 12 Revolution of 1989 Bd., 300040 Timisoara, Romania; 2 Department of Internal Medicine, Victor Babes University of Medicine and Pharmacy, 2 Eftimie Murgu Sq., 300041 Timisoara, Romania; 3 Department of Radiology and Medical Imaging, Victor Babes University of Medicine and Pharmacy, 300041 Timisoara, Romania; 4 Department of Cardiology, Victor Babes University of Medicine and Pharmacy, 2 Eftimie Murgu Sq., 300041 Timisoara, Romania; 5 Timisoara Municipal Clinical Emergency Hospital, 2A Hector Str., 300054 Timisoara, Romania

**Keywords:** metabolic syndrome, diabetes mellitus, non-alcoholic liver disease, strain imaging, liver elastography, subclinical cardiac dysfunction

## Abstract

*Background and Objectives:* Diabetes mellitus (DM) is connected to both cardiovascular disease and non-alcoholic fatty liver disease (NAFLD), and is an important component of metabolic syndrome (MetS). NAFLD can be detected and quantified using the vibration controlled transient elastography (VCTE) and the controlled attenuation parameter (CAP), whereas traditional and two-dimensional speckle tracking echocardiography (2D-STE) can reveal subclinical abnormalities in heart function. We sought to see if there was a link between left cardiac dysfunction and different levels of hepatic fibrosis in MetS patients with DM and NAFLD. *Patients and Methods:* We recruited successive adult subjects with MetS and a normal left ventricular ejection fraction, who were divided into two groups according to the presence or absence of DM. The presence of NAFLD was established by CAP and VCTE, while conventional and 2D-STE were used to assess left heart’s systolic and diastolic function. The mean age of the MetS subjects was 62 ± 10 years, 82 (55%) were men. The distribution of liver steatosis severity was similar among diabetics and non-diabetics, while liver fibrosis grade 2 and 3 was significantly more frequent in diabetics (*p* = 0.02, respectively *p* = 0.001). LV diastolic dysfunction was found in 52% of diabetic and in 36% of non-diabetic MetS patients (*p* = 0.04). 2D-STE identified in the diabetic subjects increased LA stiffness (40% versus 24%, *p* = 0.03) and reduced global left ventricular longitudinal strain (47% versus 16%, *p* < 0.0001). Liver fibrosis grade ≥ 2 was identified as an independent predictor of both subclinical LV systolic dysfunction and of LA dysfunction in MetS patients with DM (*p* < 0.0001). *Conclusions:* The current investigation confirms the link between liver stiffness and subclinical cardiac dysfunction as detected by 2D-STE in MetS patients with DM. The novel parameters derived from LA and LV 2D-STE have demonstrated greater sensitivity compared to the older measurements, and a substantial connection with hepatic fibrosis.

## 1. Introduction

Metabolic syndrome (MetS) represents a combination of many cardiovascular risk factors (insulin resistance, hypertension, hyperglycemia, central obesity, dyslipidemia). It is often associated with sedentary people [1] and has been linked to an increased risk of developing cardiovascular disease and heart failure (HF) [1,2,3,4]. Diabetes mellitus, a major component of MetS, is linked to morphologic and functional cardiac abnormalities that enhance the risk of cardiovascular atherosclerotic disease [5,6]. Early detection of subtle left cardiac dysfunction would indeed allow better risk evaluation of cardiovascular disease (CVD) in MetS patients with diabetes mellitus and improve prognosis through timely lifestyle changes and pharmacologic treatment that could stop or postpone the onset of HF.

The majority of the shortcomings of traditional echocardiogram measures are overcome by speckle-tracking echocardiography (STE), which enables direct and angle-independent analysis of myocardial deformation and hence provides sensitive and repeatable markers of myocardial fiber deformation [7]. Recent studies [8,9,10,11,12] have verified the viability and reproducibility of STE for the investigation of left atrial (LA) and left ventricular (LV) mechanics.

Due to the increasing frequency of the MetS, non-alcoholic fatty liver disease (NAFLD) became a widespread medical issue. With no alcohol abuse or other possible triggers, this illness is characterized by lipids storage of lipids, primarily triglycerides, in more than 5% of the hepatocytes. Uncomplicated steatosis is a benign condition without damage of the liver cells, inflammation, fibrosis, and represents a component of NAFLD, as is nonalcoholic steatohepatitis. According to a recently released meta-analysis, cardiac causes account for the majority of NAFLD patients’ deaths rather than liver disease complications [13].

NAFLD and cardiac dysfunction may be related, according to several research [14,15]. However, the relationship between NAFLD severity as determined by controlled attenuation parameter (CAP) and liver vibration controlled transient elastography (VCTE), and echocardiography-assessed left cardiac function has not yet been investigated.

In the current investigation, we sought to determine if left cardiac subclinical dysfunction and varying degrees of NAFLD in MetS patients with diabetes mellitus are linked in some way.

## 2. Materials and Methods

Subject selection. The departments of cardiology and internal medicine at the Victor Babes University of Medicine and Pharmacy in Timisoara, secondary medical care centers, conducted this observational research from January 2021 to August 2022. We recruited successive adult patients with MetS and LV ejection fraction (EF) ≥ 50% who agreed to have liver elastography performed as part of their assessment. They were split into two groups based on whether or not they had diabetes mellitus. In addition to 2D—traditional and STE of the left atrium and left ventricle, all individuals underwent VCTE and CAP.

Participants had to be at least 18 years old and have MetS in order to be considered for inclusion in the study. Exclusion criteria included: known coronary artery disease, peripheral artery disease, stroke, atrial flutter/fibrillation, HF with reduced (<50%)/preserved (≥50%) LVEF, NT-proBNP levels ≥ 220 pg/mL [16], cardiomyopathies; cardiac pacemakers, significant valvulopathies; chronic renal failure; chronic hepatic disease as result of drug use, viral infections, or alcohol abuse (>30 g/day in men, >20 g/day in women), serious systemic diseases or cancer.

Before the cardiac and liver ultrasound investigations, all patients underwent a clinical history analysis, a complete physical examination, a 12-lead resting electrocardiogram, and laboratory testing.

MetS was defined by the 2006 IDF parameters as central obesity (waist circumference ≥ 80 cm in women, ≥94 in males), along with just any two of the following requirements: systolic blood pressure of at least 130 mmHg or diastolic blood pressure of at least 85 mmHg, or treatment of hypertension [17], fasting plasma glucose ≥100 mg/dL or treatment of diabetes mellitus; triglyceride level ≥ 150 mg/dL or treatment for this lipid disorder; high-density lipoprotein cholesterol level < 40/50 mg/dL (men/women) [17].

Diabetes was diagnosed when the fasting plasma glucose exceeded 126 g/mL, twice in two nonconsecutive days, when glycated hemoglobin (HbA1c) was ≥6.5% or when the patient was receiving an oral hypoglycemic medication and/or insulin [18]. The patients with prediabetes (fasting plasma glucose 100–126 g/mL and HbA1c 5.7–6.4%) were included in the non-diabetic group [17].

Hypertension was diagnosed when blood pressure was ≥140/90 mmHg and/or the patient took hypertension medication [18].

### 2.1. Vibration Controlled Transient Elastography (VCTE) and Controlled Attenuation Parameter (CAP) Determinations

The same investigator performed VCTE after a fast of more than 4 h using a Fi-broScan^®^ instrument (EchoSens, Paris, France). The 3.5 MHz or 2.5 MHz probe was employed in accordance with European standards [19]. The CAP cut-offs used to differentiate the grades of steatosis were: S1 (mild)—274 dB/m, S2 (moderate)—290 dB/m, and S3 (severe)—302 dB/m [19]. In each patient, the examiner took 10 liver stiffness measures (LSM), and the median value was calculated. Measurements with a median value and an interquartile range interval/median ratio < 30% were considered valid [20]. The LSM was expressed in kilopascals (kPa). The VCTE cut-offs used to separate the degrees of fibrosis were: F2: 8.2 kPa, F3: 9.7 kPa, and F4: 13.6 kPa [21].

The same researcher used a VIVID 5S, G.E. ultrasonic phased array scope with a 3.5 MHz probe for conventional echocardiography. The diameters and volumes of the heart chambers were assessed in accordance with the recommendations of the American Society of Echocardiography [22]. The LV and LA volumes were computed using the biplane Simpson technique from the 4- and 2-chamber apical images. The LV ejection fraction (EF) was determined using the method Simpson. The diastolic function of the LV was assessed using pulsed Doppler echography in the 4- and 2-chamber apical views, with the sample volume placed at the extremity of the mitral valves. At the termination of the T wave, preceding the mitral valves opening, the maximal LA volume (LAVmax)was documented in the apical 4- and 2-chamber incidences. The minimal LA volume (LAVmin) was assessed in the early stages of ventricular diastole, after the QRS complex, as soon as the mitral valves closed. The total LA stroke volume (tLASV) was calculated as the difference between LAVmax and LAVmin. The LA ejection fraction (EF, %) was estimated using the formula 100× [LAVmax-LAVmin]/LAVmax [23,24].

**Figure 1:**
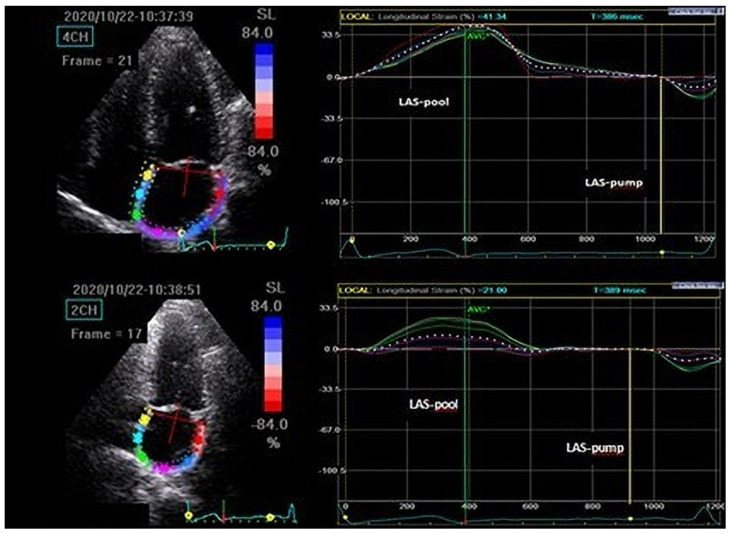
Left atrial two-dimensional speckle tracking echocardiography. Abbreviation: LAS, left atrium strain.

*The 2D-speckle tracking echography (2D-STE)* was performed utilizing the Vivid Echo PAC software 201 (GE Medical System) at 60 to 90 frames/s. At least three successive cardiac cycles were captured for the duration of a breath-hold in 4- and 2-chamber apical views. The video analysis was completed offline. The atrial endocardium and epicardium were tracked mechanically and afterwards adjusted by the examiner. The software split the atrium into six regions. The peak LA-pool strain was measured just before the mitral valve opening, and the peak LA-pump strain just before the P wave (Figure 1). LA stiffness was estimated as the E/A value divided by the peak LA-pool strain [25,26].

**Figure 2:**
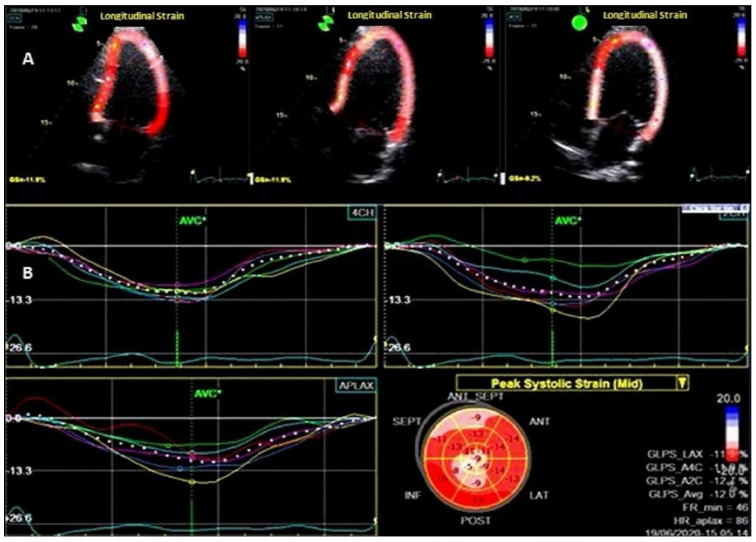
Left ventricular two-dimensional speckle tracking echocardiography. (**A**) Apical 2, 3, and 4 chamber incidences longitudinal strain analysis; (**B**) Bull’s eye synopsis.

For the study of ventricular myocardial deformation, the Echo PAC software 201 was set at a frequency of 70 to 80 frames/s [23,27]. The program split the ventricle into six parts (Figure 2). These 6 segments’ 2D-ST images were examined in 4, 3, and 2-chamber apical incidences. The average of the values recorded in the 18 investigated segments was used to compute the peak global longitudinal strain (GLS).

Patients with poor echocardiographic image resolution were excluded from the research.

The cut-off parameters for LA dysfunction were: <50% LA ejection fraction, <30% LA pool strain, <8% LA pump strain, and ≥0.38 LA stiffness [28,29]. E/A ratio < 0.8 and IVRT > 100 ms were the defining criteria for LV diastolic dysfunction; FEVS < 50% and peak GLS- < −18% for LV systolic dysfunction [23,27].

#### 2.1.1. Ethics

All research participants provided written informed consent. The study was carried out in accordance with the Helsinki Declaration’s principles and was authorized by the Ethics Committee of the “Victor Babeș” University of Medicine and Pharmacy Timișoara (nr. 1a/16 Jane 2016).

#### 2.1.2. Statistical Analysis

The MedCalc^®^ Statistical Software 20.210 version was used for statistical analysis (MedCalc Software Ltd., Ostend, Belgium). The mean and standard deviation (SD) for continuous data were presented. Numbers and percentages were used to represent categorical variables. The paired t-test was used to compare the differences between the groups. Pearson’s correlation coefficient was used to assess the relationship between variables. The variables associated with LA and LV dysfunction were addressed to univariate and multivariate logistic regression analysis. For all tests, *p* < 0.05 was regarded as statistically noteworthy.

#### 2.1.3. Reproducibility

The research was carried out by the same echocardiographer, respectively by the same liver sonographer. The intra-class correlation coefficient (ICC) was calculated for intra-observer reproducibility and revealed high intra-observer conformity. For echocardiography the ICC was 0.86 (95% CI 0.79–0.91), and 0.88 (95% CI 0.79–0.92) for hepatic elastography.

## 3. Results

Of the 212 MetS participants primarily studied, 38 (18%) were excluded due to poor resolution of echocardiographic images, and 28 (13%) were withdrawn due to validation failures at CAP and VCTE.

Finally, 150 MetS subjects were included in the research and were divided into two groups based on the existence of diabetes mellitus. Table 1 shows the demographic and clinical characteristics of the two groups. The mean age range of the subjects was 62 ± 10 years (31–79 years). Males made up 82 of the participants (55%). There were no significant differences among the groups regarding age and gender distribution, hypertension prevalence, waist circumference, body mass index, smoking status, number of MetS components, and medication for lipid disorders and hypertension. The differences between the levels of serum transaminases, total cholesterol, low density and high-density cholesterol lipoproteins, and pro NT-brain natriuretic peptides were also not notable. Diabetics had increased triglyceride, glycosylated hemoglobin, and fasting plasma glucose levels.

The diabetic MetS subjects presented the following dispersal of steatosis severity assessed by CAP: 8 (11%)—S1, 25 (35%)—S2, and 39 (54%)—S3. S2 and S3 were significantly more frequent than in non-diabetic MetS patients, as shown in Table 2. The distribution of fibrosis stages F2 and F3 was also notably higher in diabetic versus non-diabetic patients (*p* = 0.02 and *p* = 0.001, respectively).

Table 3 displays echocardiography data. There were no considerable differences in the traditional measurements of LV function and structure between the two groups, with the exception of LV diastolic dysfunction, which was more common in diabetes subjects. We also discovered no alterations in LA diameters, volumes, or ejection fractions between diabetic and non-diabetic MetS patients.

The 2D-STE, on the other hand, detected subtle LV systolic and subtle LA dysfunction in the diabetic patients, revealed by significant lower LV global longitudinal strains, decreased LA reservoir and pump strains, as well as by increased LA stiffness.

LV diastolic dysfunction, identified by traditional echocardiography, was found in 38 diabetic (53%) and in 40 (51%) nondiabetic patients, *p* = 0.04. Subtle LV systolic dysfunction, defined by GLS < 18%, was found in 47 (65%) diabetic patients and in 34 (44% non-diabetic subjects *p* < 0.0001). Subtle LA dysfunction, identified by LA stiffness > 0.38 was found in 29 (40%) diabetic MetS patients and in 15 (19%) non-diabetic patient, *p* = 0.03. The diabetic MetS patients had a 1.5 times higher risk of LV systolic dysfunction (95% CI 1.10 to 2.02, *p* < 0.01), and a two-fold higher risk of LA dysfunction (95% CI 1.22 to 3.57, *p* < 0.01)

Both in univariate and in multivariate logistic regression analysis, liver fibrosis ≥ 2 was an independent predictor of subtle LV and subtle LA dysfunction in diabetic MetS patients with NAFLD (Table 4).

**Figure 3:**
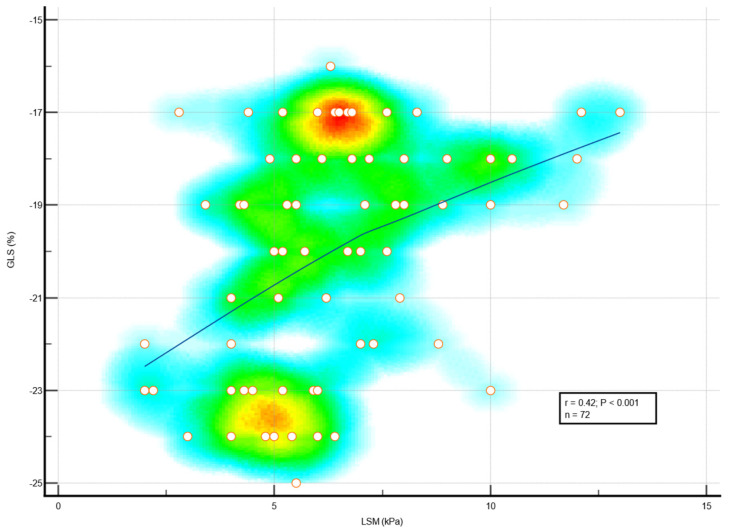
Correlation between LSM and global LV longitudinal strain in MetS patients with diabetes. Abbreviations: GLS, global longitudinal ventricular strain; LSM, liver stiffness measurement; MetS, metabolic syndrome.

**Figure 4:**
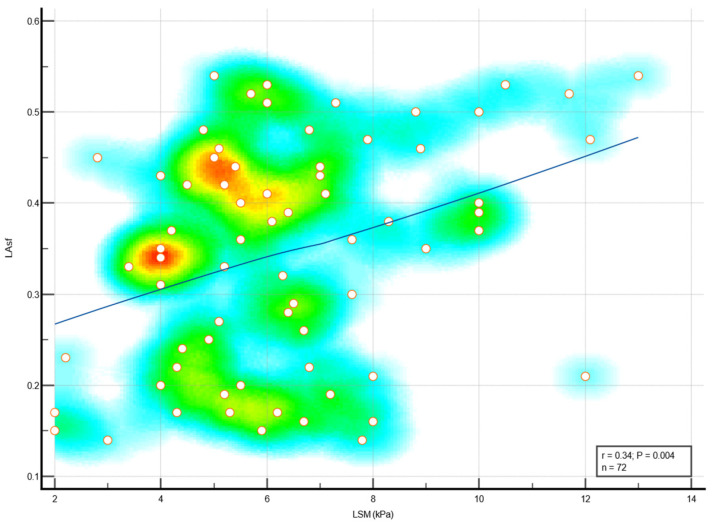
Correlation between LSM and LA stiffness in diabetic MetS subjects. Abbreviations: MetS, metabolic syndrome; LAsf, left atrial stiffness; LSM, liver stiffness measurement.

We found significant associations between liver fibrosis ≥ 2 with both subtle LV systolic dysfunction and subtle LA dysfunction in diabetic MetS patients. Subclinical LV systolic dysfunction, assessed by reduced global longitudinal strain (%), was significantly linked with liver stiffness (kPa), *p* < 0.001 (Figure 3). LA dysfunction, assessed by increased LA stiffness (%) was also significantly associated with liver stiffness (kPa), as shown in Figure 4.

## 4. Discussion

MetS has gained a significant interest in the past few decades as a result of its rising incidence rate among the overall population. MetS affects approximately one-fourth of the world’s population [2], and it is anticipated that the number of people affected by MetS will continue to rise in line with the incidence of obesity and type 2 diabetes. In general, the research have indicated that persons with MetS are at a greater risk of suffering CVD events. The largest systematic review and meta-analysis (n = 951,083) established a double increase in the risk of cardiovascular disease morbidity and mortality, and a 1.5-fold increase in the risk of all-cause mortality associated with MetS [1].

In medical practice, NAFLD constitutes a large segment of liver disease. The general population has a NAFLD prevalence of 10–30%, with greater rates in industrialized and developing nations. NAFLD has been linked to insulin resistance and the metabolic syndrome. One probable explanation for this connection is that NAFLD patients frequently have abnormal glucose metabolism, are overweight or obese, and have hypertension [30].

Patients with NAFLD have a much greater risk of dying from ischemic cardiovascular disease (12% to 16%) than those without this condition (1% to 3%) [15,28]. This finding suggests a substantial link between the level of severity of NAFLD and the chance of dying from cardiovascular disease. This connection might be explained by a variety of processes, one of which being insulin resistance [3]. Cardiac insulin resistance leads to altered energy absorption, since the heart consumes energy from glucose and fatty acids [31]. Changes in myocardial structure and function are a result from changed energy absorption.

MetS patients share the same altered myocardial energy uptake mechanism. Obesity, high blood pressure, and diabetes were found to cause fibrotic changes in the LV and LA ventricular myocardial walls [32,33]. As myocardial fibrosis progresses, LV diastolic compliance declines and LV filling pressures rise, both of which exert an effect on LV and LA function.

The most serious diabetes-related complications and the leading cause of death in diabetic patients are cardiac complications [34], with evidence indicating a wide range of changes in diabetic hearts, including cardiomyocyte hypertrophy, myocardial interstitial fibrosis, and cardiomyocyte apoptosis [8,35,36]. It has been demonstrated that functional remodeling occurs before structural remodeling, resulting in dilatation of the heart chambers.

Our study included MetS adults, with and without diabetes mellitus, without established cardiovascular disease. No subject had LV heart failure, neither with reduced, or with preserved LVEF, according to the clinical examination records, conventional echocardiography data, and the NT-proBNP levels.

Participants in our study were thoroughly examined utilizing liver VCTE and CAP to identify and quantify NALD, along with conventional and speckle-tracking echography to evaluate cardiac structure and function.

Liver steatosis grade S2 and S3 were significantly more frequent than in non-diabetic MetS patients, as shown in Table 2, as well as liver fibrosis F2 and F3.

Although the LA and LV diameters, volumes, and ejection percentages did not differ substantially between MetS participants with and without DM, the 2D-STE revealed LA and LV deformation features that were significantly worse in the presence of DM, indicating subclinical dysfunction (*p* < 0.04). According to previous research, the pattern of myocardial deformation is highly related to the severity of myocardial fibrosis determined by cardiac MRI or histopathologic samples [37].

Despite evidence that patients with NAFLD are at risk of cardiac structural abnormalities, no link between myocardial and hepatic fibrosis has been demonstrated. This is the first study to show this link using the deformation patterns of the left heart in diabetics with MetS. In multivariate regression analysis, liver fibrosis grade ≥ 2 was significantly associated with LA stiffness ≥ 0.38 (*p* < 0.0001), and with subclinical LV systolic dysfunction (*p* < 0.0001) in MetS patients with NAFLD and DM.

Early diagnosis of heart and liver dysfunction in MetS patients with diabetes is crucial because adequate lifestyle changes and pharmacological therapy may be able to prevent or postpone the onset of heart failure and liver cirrhosis, respectively. These approaches have the potential to reduce morbidity and mortality rates, as well as the costs of providing health care [38,39].

The fact that patients with prediabetes (fasting plasma glucose 100–126 g/mL and HbA1c 5.7–6.4%) were included in the non-diabetic group” might have influenced somehow the results of the study.

*Limitations.* All patients had their liver steatosis and fibrosis assessed non-invasively, without performing a liver biopsy, which is the gold standard procedure. We did not analyze in a separate group the MetS subjects with prediabetes. They were included in the nondiabetic group.

## 5. Conclusions

Our findings imply that liver assessment by CAP and VCTE, as well as heart function assessment by 2D-STE, should be routinely done in diabetic individuals with MetS, as they might detect subclinical abnormalities. The innovative parameters derived from the LA and LV deformation patterns demonstrated to be more sensitive than the traditional ones, and were found to be significantly linked with liver stiffness in these subjects.

## Figures and Tables

**Table 1:** Clinical and laboratory features of MetS patients.

	With DM (n = 72)	Without DM (n = 78)	*p* Value
Age (years)	62.8 ± 8.7	60.2 ± 10.7	0.31
Male sex n (%)	38 (53)	44 (56)	0.71
Systemic hypertension (n, %)	62 (80%)	58 (84%)	0.52
Smoking (current, %)	7 (9%)	8 (10%)	0.83
Systolic BP (mmHg)	142.9 ± 15	140.1 ± 19	0.32
Diastolic BP (mmHg)	85.4 ± 11	83.7 ± 11	0.34
Heart rate (beats/min)	77.4 ± 11.3	73.9 ± 11.1	0.05
BMI (kg/m^2^)	33.6 ± 5.3	32.0 ± 4.8	0.05
Waist circumference (cm)	114 ± 12	110 ± 13	0.05
Total cholesterol (mg/dL)	174 ± 40	183 ± 62	0.24
HDL (mg/dL)	44.4 ± 13.7	45.7 ± 11.8	0.53
LDL (mg/dL)	129.4 ± 31	130.5 ± 30	0.82
Triglyceride (mgl/dL)	172.7 ± 109	134.4 ± 80.4	**0.01**
FPG (mg/dL)	153 ± 50	108 ± 11	**<0.0001**
HbA1c (%)	7.1 ± 0.9	5.2 ± 0.7	**<0.0001**
ASAT (U/L)	24 ± 9	23 ± 5	0.39
ALAT (U/L)	37 ± 7	36 ± 5	0.31
NT-proBNP (pg/mL)	144 ± 66	129 ± 65	0.16
Number of MetS components	3.9 ± 0.8	3.7 ± 0.6	0.08
Number of components ≥ 4	39 (54%)	34 (44%)	0.22
Duration of diabetes (years)	6.5 ± 4	-	-
Diabetes treatment Oral antidiabetics Insulin	51 (70%) 18 (25%)	-	-
Statins	49 (68%)	57 (73%)	0.50
Fibrates	21 (29%)	17 (21%)	0.25
ACEI/ARB	35 (49%)	43 (55%)	0.46
Beta-blockers	55 (76%)	58 (74%)	0.77
Calcium antagonists	40 (56%)	43 (55%)	0.90
Diuretics	52 (72%)	55 (70%)	0.79

Notes: Data are given as a mean, standard deviation, or as number, percentage. Statistically notable values (*p* < 0.05) are shown in bold. Abbreviations: ALAT, alanine amino transferase; ASAT, aspartat amino transferase; BP, blood pressure; BMI, body mass index; HbA1c, glycosylated hemoglobin; FPG, fasting plasma glucose; LDL, low density lipoprotein; HDL, high density lipoprotein; MetS, metabolic syndrome; NT-proBNP, N-terminal pro- brain type natriuretic peptides; U, units; ACEI, angiotensin converting enzyme inhibitors; ARB, angiotensin-1 receptor blockers.

**Table 2:** Assessment of hepatic fibrosis and steatosis.

	With DM (n = 72)	Without DM (n = 78)	*p* Value
CAP, dB/m	292.42 ± 43.6	299.36 ± 60.7	**<0.0001**
Steatosis stage			
S0	0 (0%)	0 (0%)	**1**
S1	8 (11%)	37 (47%)	**<0.0001**
S2	25 (35%)	14 (18%)	**<0.02**
S3	39 (54%)	27 (35%)	**<0.02**
LSM, kPa	11.53 ± 5.94	8.00 ± 3.58	**<0.0001**
Fibrosis stage			
F0-1	27 (38%)	57 (73%)	<0.0001
F2	28 (39%)	17 (22%)	0.02
F3	15 (21%)	3 (4%)	0.001
F4	2 (2%)	1 (1%)	0.61

Notes: Data are given as a mean, standard deviation, or as number, percentage. Statistically important values (*p* < 0.05) are presented in bold. Abbreviations: F, fibrosis; S, steatosis; CAP, controlled attenuation parameterliver; LSM, stiffness measurements.

**Table 3:** LV echocardiography results in MetS patients.

	With DM (n = 72)	Without DM (n = 78)	*p* Value
Traditional echocardiography			
LV End DD (mm)	49.14 ± 3.22	48.73 ± 2.97	0.41
LV End SD (mm)	30.37 ± 2.54	29.87 ± 2.72	0.24
LA diameter (mm)	3.14 ± 0.33	3.24 ± 0.35	0.07
LVEF (%)	51.5 ± 0.6	51.6 ± 0.5	0.26
LVFS (%)	37.99 ± 2.93	38.10 ± 3.55	0.83
LV diastolic dysfunction (n, %)	38 (52%)	28 (36%)	**0.04**
LA volumes (mL)			
Maxim	27.4 ± 5.3	26.9 ± 5.5	0.57
Minim	11.55 ± 4.0	12.56 ± 3.7	0.11
LA ejection fraction (%)	56.2 ± 4.2	56.9 ± 3.8	0.28
2D-STE			
LAS-pool (%)	43.9 ± 4.3	45.4 ± 3.7	**0.02**
LAS-pump (%)	17.4 ± 2.2	18.3 ± 1.8	**<0.01**
LA stiffness	0.34 ± 0.12	0.28 ± 0.15	**<0.01**
LAsf ≥ 0.38	29 (40%)	19 (24%)	**0.03**
GLS (%)	20.1 ± 2.4	21.6 ± 2.1	**0.0001**
GLS ≤ 18%	34 (47%)	13 (16%)	**<0.0001**

Notes: Data are given as a mean, standard deviation, or as number, percentage. Statistically important values (*p* < 0.05) are presented in bold. Abbreviations: GLS, global longitudinal strain. MetS, metabolic syndrome; DM, diabetes mellitus; EF, ejection fraction; End SD, end systolic diameter; End DD, end diastolic diameter; FS, fractional shortening; LV, left ventricle; LA, left atrium; 2D-STE, two dimensional speckle tracking echography; LAS, left atrial longitudinal strain.

**Table 4:** Independent predictors of LV systolic dysfunction in MetS patients with DM.

Parameter	Univariate Analysis			Multivariate Analysis		
	β	SE	*p*	β	SE	*p*
Liver steatosis ≥ S2	2.17	0.62	**<0.001**			
Liver fibrosis ≥ F2	3.60	0.81	**<0.0001**	3.14	0.43	**<0.0001**
**Independent predictors of LA stiffness ≥ 0.38 in MetS patients with DM**						
**Parameter**	**Univariate analysis**			**Multivariate analysis**		
	**β**	**SE**		**β**	**SE**	
Liver fibrosis ≥ F2	3.60	0.81	**<0.0001**	3.14	0.43	**<0.0001**

Notes: Statistically notable values are presented in bold (*p* < 0.05). The adjusted parameter in multivariate analysis was diabetes mellitus. Abbreviations: LV, left ventricle; LA, left atrium; MetS, metabolic syndrome; DM, diabetes mellitus.

## Data Availability

Not applicable.
